# Ecthyma Gangrenosum Caused by Streptococcus pyogenes: A Case Report

**DOI:** 10.7759/cureus.111039

**Published:** 2026-06-17

**Authors:** Kiley B Kirby, John C Holman, Moses H Cheng

**Affiliations:** 1 Department of Family Medicine, Naval Hospital Camp Pendleton, Oceanside, CA, USA

**Keywords:** ecthyma gangrenosum, ecthyma gangrenosum (eg), group a strep sepsis, group a streptococcus pyogenes, immunocompromised patient, non pseudomonal ecthyma gangrenosum, streptococcus pyogenes infection, type 1 diabetes mellitus (t1d)

## Abstract

Ecthyma gangrenosum (EG) is an uncommon infectious vasculitis presenting as erythematous macules that develop into characteristic black eschars most commonly in the setting of bacteremia. While EG is most often associated with *Pseudomonas aeruginosa* infection, we present a case of EG-like skin lesions caused by *Streptococcus pyogenes*, an atypical causative organism, in an immunocompromised individual. Resolution was achieved following treatment with disease-specific intravenous antibiotics, adjuvant topical therapies, and stabilization of other underlying medical conditions that precipitated the infection.

## Introduction

Ecthyma gangrenosum (EG) is an infectious vasculitis caused by bacterial occlusion and destruction of small vessels, leading to subsequent cell death. It typically occurs in the setting of systemic infection, most commonly affecting the anogenital region, intertriginous areas, and the extremities. It was first characterized in 1897 by Canadian pathologist Dr. Lewellys Barker secondary to a *Pseudomonas aeruginosa* infection [1-3]. Historically, EG has been understood to be pathognomonic for *P. aeruginosa* septicemia; however, atypical causes are being cited more commonly in the literature, broadening the understanding of the disease presentation to include non-pseudomonal EG, also defined as EG-like skin lesions [1]. The disease process commonly occurs in immunocompromised individuals or those with conditions that allow for increased infectious susceptibility, such as neutropenia, underlying malignancies, or other immunocompromising states [1].

Ecthyma gangrenosum initially presents as painless erythematous or purpuric macules that develop vesicles or bullae, progressing to a necrotic ulcer with a distinct black eschar. While diagnosis of EG is generally made based on characteristic clinical appearance, typical histological characteristics of EG include invasion of the dermis and blood vessels by the causative organism, epidermal and dermal necrosis, and thrombus [1,4].

Treatment of EG is comprised of organism-specific antibiotic treatment resulting in improvement of cutaneous findings, and surgical debridement may be required in the setting of an abscess, rapid spread of necrotic tissue, or poor response to antibiotic therapy. Residual scarring can be seen and usually correlates with disease severity, size of cutaneous lesions, partial versus full thickness necrosis, healing via primary or secondary intention, and other non-modifiable and modifiable risk factors [5,6].

## Case presentation

A 33-year-old non-verbal male with Trisomy 18 and poorly controlled type 1 diabetes mellitus (T1DM) presented to a California emergency department (ED) accompanied by his caregivers following three days of fever, increased urine output, and worsening, painful bilateral upper-extremity lesions. Caregivers reported no known preceding trauma, significant environmental or animal exposures, or travel. Prior to presentation, the patient was only treated with over-the-counter analgesia with minimal benefit.

**Figure 1:**
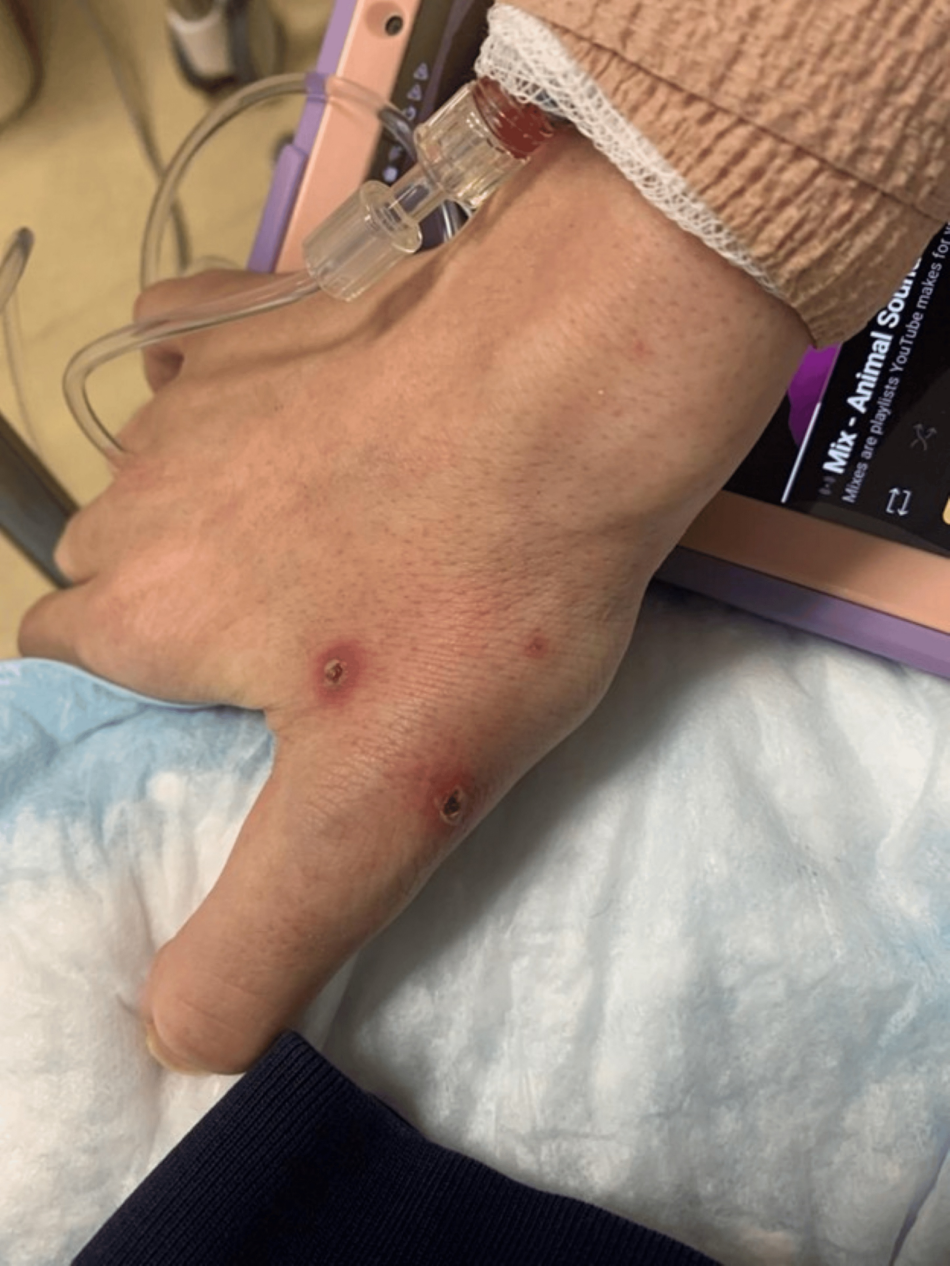
Dorsal aspect of the right pollex demonstrating two black eschars surrounded by erythematous halos

**Figure 2:**
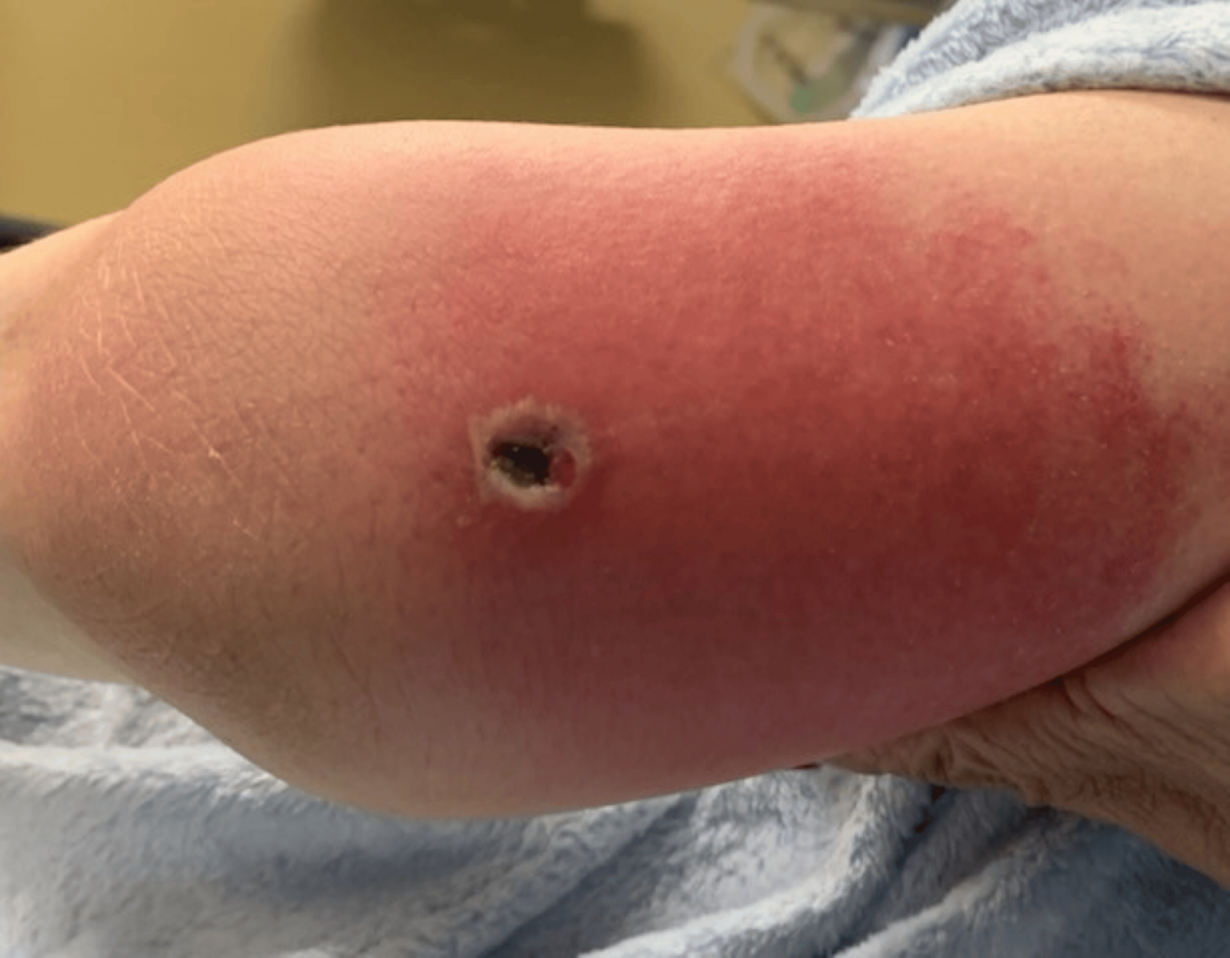
Posterior left upper extremity with a single black eschar surrounded by a patch of surrounding erythema

**Figure 3:**
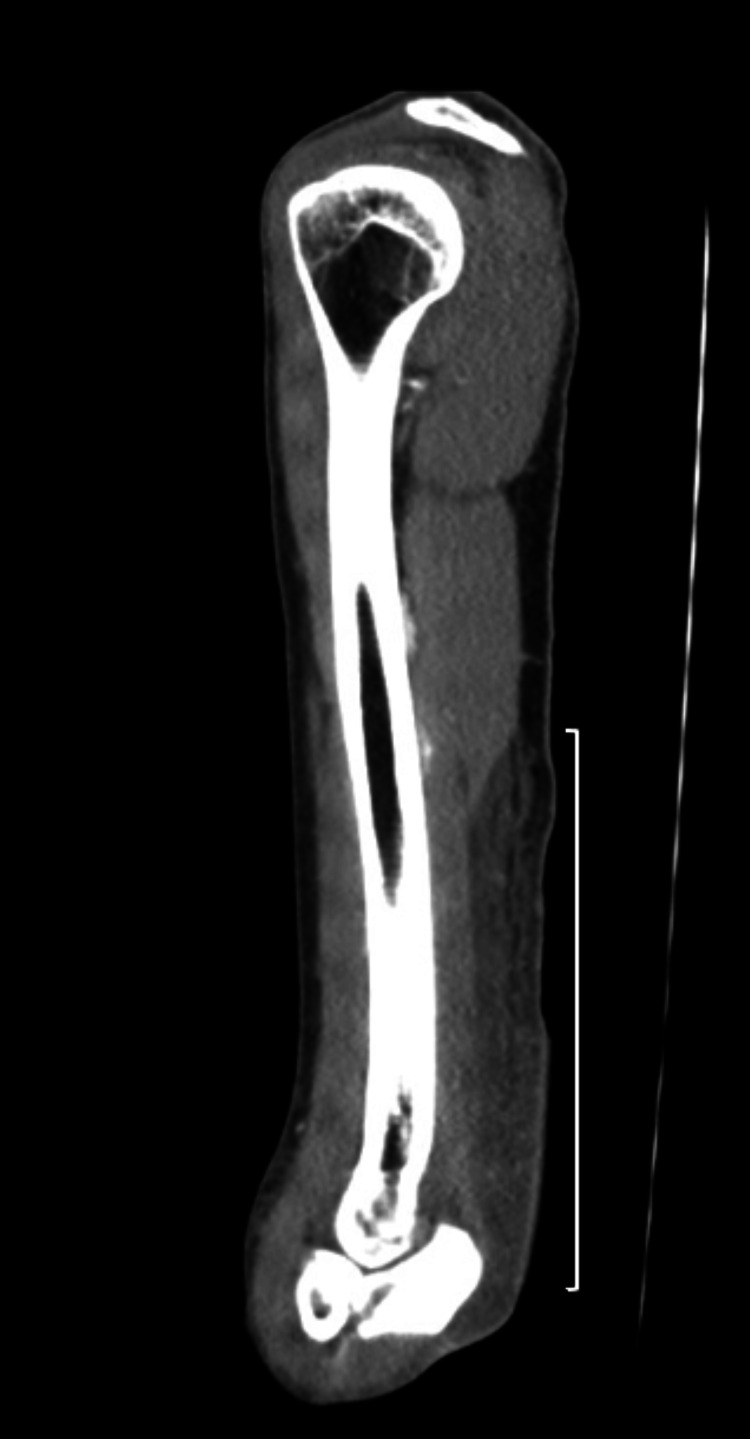
A CT scan of the left upper extremity in the sagittal plane showing subcutaneous thickening with no evidence of defined fluid collection, abscess, or subcutaneous gas extending from the level of the mid left humerus posteriorly and medially through the left elbow as outlined by the bracket on the right

Vital signs were significant for fever and tachycardia. Physical exam revealed dry mucous membranes, delayed capillary refill, an established grade IV holosystolic murmur, and erythematous patches containing central ulcerations with black eschars on the dorsal aspect of the right pollex (Figure 1) and posterior left upper extremity (Figure 2). Laboratory analysis revealed elevated white blood cell count, hyperglycemia with low bicarbonate, and an elevated anion gap consistent with severe diabetic ketoacidosis (DKA). The patient also had a significantly elevated hemoglobin A1c (Table 1). A CT scan of the left upper extremity contained evidence of skin thickening and subcutaneous fluid correlating to the area beneath the visible erythema, but was without a defined collection or abscess, osteomyelitis, or gas in the soft tissues suggestive of necrotizing fasciitis (Figure 3). Blood cultures were obtained in the ED before starting empiric antibiotics. 

**Table 1:** Summary of key laboratory findings at initial presentation

Test	Result	Reference range
White blood cell count	24.3 10*3/uL	4.5-11.0 10*3/uL
Carbon dioxide	20 mmol/L	22-29 mmol/L
Glucose	477 mg/dL	74-106 mg/dL
Anion gap	18 mEq/L	6-16 mEq/L
Hemoglobin A1c	10.8%	4.8-5.6%

The patient was admitted to the intensive care unit for management of DKA with intravenous (IV) fluids and an insulin drip. Broad-spectrum antibiotics were initiated and subsequently narrowed to IV Penicillin G following isolation of *Streptococcus pyogenes*, or Group A Strep (GAS), in the initial blood cultures, which were only processed anaerobically. Subsequent aerobic and anaerobic cultures were negative for the remainder of his hospital course. The patient’s murmur remained unchanged during hospitalization, and he remained without development of clinical manifestations of infectious endocarditis. A transthoracic echocardiogram was completed eight days following the initial positive culture without evidence of cardiac involvement. The infectious disease service recommended continued targeted therapy for 14 days from the first negative blood cultures. The patient and caregivers were offered midline catheter placement to allow for at-home treatments, but they elected to remain admitted to the hospital for completion of the IV antibiotic course.

The patient required a total of 16 days of IV penicillin-G prior to discharge, with improvement in cutaneous findings by hospital day 4 precluding the need for surgical debridement. A dermatology consultation recommended adjuvant therapy with topical tacrolimus and zinc oxide, which was initiated following nine days of negative blood cultures and clinical improvement of lesions for treatment of post-inflammatory erythema. The one-week follow-up post-discharge revealed well-healing lesions without surrounding erythema or swelling.

## Discussion

This case highlights a manifestation of non-pseudomonal EG-like skin lesions precipitated by an atypical causative organism in a susceptible host. The patient was diagnosed with non-pseudomonal EG based on the characteristic black eschars surrounded by tender halos and patches of erythema in the setting of *S. pyogenes* bacteremia. While there have been previous reports in the literature of EG caused by *S. pyogenes* and other gram-positive organisms, it remains an uncommon isolate. Historically associated with *P. aeruginosa*, which has an established propensity to cause vascular dysfunction and angioinvasion, other causative organisms of EG have been documented in the literature (Table 2) [7]. The connection among these published cases is not pathogen-specific but pathologically concordant, characterized by dermal, epidermal, and vascular dysfunction that leads to tissue necrosis and vascular thrombus, resulting in classic skin findings.

**Table 2:** Summary of previously published literature reviews and case reports of EG EG: Ecthyma gangrenosum

Literature	No. of cases referenced	Categorization of organism	Isolated causative organism	Reason for immunosuppressive state	Additional notes	
Vaiman et al. (2014) [8]	167 (71% total cases)	Gram-negative bacteria	P. aeruginosa	N/A	Literature review from 1975 to 2014	
Vaiman et al. (2014) [8]	29 (18% total cases)	Gram-negative bacteria	Not specified	N/A	Literature review from 1975 to 2014	
Vaiman et al. (2014) [8]	15 (11% total cases)	Fungal	Not specified	N/A	Literature Review from 1975 to 2014	
Reich et al. (2004) [9]	One	Gram-negative bacteria	Citrobacter freundi	Acute myeloid leukemia (AML)	Initially non-bacteremic EG that progressed to bacteremia	
Alkan (2023) [3]	One	Gram-negative bacteria	Achromobacter xylosoxidans	Undergoing chemotherapy treatment	Patient died from sepsis-related complications	
Koumaki et al. (2019) [10]	One	Gram-negative and Gram-positive bacteria	*Klebsiella pneumoniae* and *Streptococcus vestibularis*	AML	Polymicrobial with both gram-positive and negative organisms	
Yanagi et al. (2022) [11]	One	Gram-positive bacteria	*S. pyogenes* (GAS)	Recent organ transplantation	Same causative organism as represented in the presented case	
Trillig et al. (2019) [12]	Two	Gram-positive bacteria	*S. pyogenes* (GAS) and *Staph aureus*	One case with no known risk factors or immunodeficiency; one case with advanced HIV	Same causative organism as represented in presented case, polymicrobial	
Cantania et al. (2026) [13]	One	Gram-positive bacteria	Erysipelothrix rhusiopathiae	Neutropenia following infection with dengue virus	Dengue virus can cause bone marrow suppression resulting in neutropenia	
Kimyai-Asadi et al. (1999) [14]	One	Viral	Herpes simplex virus (HSV)	No known immunodeficiency	First reported case of EG caused by HSV	
Ferguson et al. (2017) [15]	Two	No isolated organism	No isolated organism	Once case with HIV; one case with Good syndrome	Typical causative organism but the causes occurred without bacteremia	

Ecthyma gangrenosum is most frequently described in the setting of bacteremia in immunocompromised hosts. However, it can occur both inside and outside the setting of bacteremia. Localized anogenital EG is often independent of systemic infection [1]. Reich et al. described a case with characteristic EG lesions in isolation with subsequent progression to bloodstream infection, describing a unique presentation of EG both with and without bacteremia [9]. In our case, the patient’s initial cultures were positive for *S. pyogenes*. However, his lesions were present prior to presentation and could have allowed for a point of inoculation, thus precipitating sepsis and progression to DKA. This patient also had evidence of poor chronic control of his T1DM at admission, which may have reduced his immune function, leading to the development of sepsis and progression to EG.

Typically, the immunosuppression precipitating EG is caused by severe neutropenia or use of immunosuppressive agents; however, it may also be caused by malnutrition, chronic disease, or genetic syndromes that affect B and T cell function [1]. Immunocompromised patients develop EG due to inadequate immune response leading to higher pathogen colonization, reduced host inhibition of virulent factors, impaired chemotaxis of immune cells, and systemic alteration of pathogen clearance [16]. In this case, the patient’s severe DKA and poorly controlled T1DM contributed to both a decrease in acute and chronic immune competency. Another complicating factor includes the patient’s history of Trisomy 18. While immune function in adult patients with Trisomy 18 is not well characterized, the underlying multisystem disease and reduced physiological reserve likely contributed to this patient’s vulnerability for disease. 

Evolution of erythematous macules into patches with a central black eschar is classic for EG; however, other mimics should be considered within the appropriate clinical context. Other etiologies considered included cutaneous anthrax and leishmaniasis, though this patient was without appreciable exposure to these pathogens. While DKA can precipitate fungal infections with opportunistic fungi such as Rhizopus, Absidia, or Mucor, disseminated dermatologic manifestations of mucormycosis characteristically follow rhino-cerebral infections and predominately affect the face and sinuses [17]. Erythema nodosum may occur in the presence of streptococcal infections; however, it is generally localized to the extensor surfaces of lower extremities and often does not result in eruption of the skin [18]. Ultimately, the characteristic cutaneous findings in the setting of established bacteremia in an immune-susceptible patient established the diagnosis of non-pseudomonal EG. Targeted treatment against the isolated organism resulted in disease remission and eventual resolution, further supporting the diagnosis without necessitating tissue biopsy.

## Conclusions

This case describes the development of non-pseudomonal EG or EG-like skin lesions in an immunodeficient patient precipitated by an atypical causative organism: S. pyogenes. While typically associated with P. aeruginosa, diverse causative pathogens are increasingly being identified and should be considered in developing pathogen-specific treatment plans for patients with EG. Recognizing the broadening definition of EG as a clinical syndrome rather than a pathogen-specific condition is essential in guiding appropriate evaluation and organism-specific management.
